# Facile Fabrication and Characterization of Improved Proton Conducting Sulfonated Poly(Arylene Biphenylether Sulfone) Blocks Containing Fluorinated Hydrophobic Units for Proton Exchange Membrane Fuel Cell Applications

**DOI:** 10.3390/polym10121367

**Published:** 2018-12-10

**Authors:** Kyu Ha Lee, Ji Young Chu, Ae Rhan Kim, Dong Jin Yoo

**Affiliations:** 1 Department of Energy Storage/Conversion Engineering of Graduate School, Hydrogen and Fuel Cell Research Center, Chonbuk National University, Jeonju 54896, Korea; carumiss@naver.com (K.H.L.); ebbuneg@hanmail.net (J.Y.C.); 2 R&D Center for CANUTECH, Business Incubation Center and Department of Bioenvironmental Chemistry, Chonbuk National University, Jeonju 54896, Korea; 3 Department of Life Science, Chonbuk National University, Jeonju 54896, Korea

**Keywords:** proton exchange membrane, fuel cell performance, small angle X-ray scattering

## Abstract

Sulfonated poly(arylene biphenylether sulfone)-poly(arylene ether) (SPABES-PAE) block copolymers by controlling the molar ratio of SPABES and PAE oligomers were successfully synthesized, and the performances of SPABES-PAE (1:2, 1:1, and 2:1) membranes were compared with Nafion 212. The prepared membranes including fluorinated hydrophobic units were stable against heat, nucleophile attack, and physio-chemical durability during the tests. Moreover, the polymers exhibited better solubility in a variety of solvents. The chemical structure of SPABES-PAEs was investigated by ^1^H nuclear magnetic resonance (^1^H NMR), Fourier transform infrared spectroscopy (FT-IR), and gel permeation chromatography (GPC). The membrane of SPABES-PAEs was fabricated by the solution casting method, and the membranes were very flexible and transparent with a thickness of 70–90 μm. The morphology of the membranes was observed using atomic force microscope and the ionic domain size was proved by small angle X-ray scattering (SAXS) measurement. The incorporation of polymers including fluorinated units allowed the membranes to provide unprecedented oxidative and dimensional stabilities, as verified from the results of ex situ durability tests and water uptake capacity, respectively. By the collective efforts, we observed an enhanced water retention capacity, reasonable dimensional stability and high proton conductivity, and the peak power density of the SPABES-PAE (2:1) was 333.29 mW·cm^−2^ at 60 °C under 100% relative humidity (RH).

## 1. Introduction

Polymer electrolyte fuel cells (PEFCs) are attracting immense attention as alternative power sources for portable and stationary electronics as they allow H_2_ and O_2_ gases to generate energy with low/zero production of poisonous gases. Among the working components in proton exchange membrane fuel cells (PEMFCs), the proton exchange membrane (PEM) is a pivotal module, which selectively transfers the protons from the anode to the cathode. Commercial perfluorinated ionomers, such as Nafion and Flemion, are being extensively exploited as PEMs because of their excellent electrochemical properties, elevated proton conductivity and good mechanical modulus [1,2,3,4]. However, the decrease of proton conductivity at high temperature under low humidity, high methanol crossover and low glass transition temperature are the inevitable confines of perfluorosulfonic acid (PFSA) membranes that complicate the practical PEMFC operation. Accordingly, wide research efforts and activities in the fabrication of alternative PEMs are thriving.

Aromatic hydrocarbon polymers, such as sulfonated polyketones (SPKs) [5], sulfonated polyimides (SPIs) [6], sulfonated poly(arylene sulfone)s (SPASs) [7,8], sulfonated poly(arylene ether sulfone)s (SPAESs) [9] are highlighted for their exclusive attributes such as low cost, high mechanical modulus resulting from their extended polymer chains, and flexible proton conductivity due to their adjustable degree of sulfonation (DS). The direct sulfonation method has some advantage such as avoiding cross-linking, other side reactions, and also providing good mechanical properties. Recently, investigators have attempted to improve the performance of proton conductivity through controlled morphology using directly sulfonated monomers rather than using a post sulfonation method. Therefore, the exploitation of aforementioned polymers for PEM preparation is flourishing. However, the polymeric membranes are susceptible to distortion due to volumetric changes caused by the water drift in the membranes. Such type of distortion can lead to increased pressure between the bipolar plates and membrane electrode assembly (MEA), resulting in the formation of crack, pinholes and perforations in the membrane. On the other side, chemical degradation of polymeric chains caused by the hydroxyl (·OH) and hydroperoxyl (·OOH) radical attacks in the cathode side, further exacerbate the cracks in the membranes. Altogether, these issues drastically decline the overall performance of PEMFC. It has been shown that incorporation of fluorinated segments into the polymer chains is an ultimate strategy in improving the mechanical modulus, chemical stability, and fuel barrier properties of PEMs, which enhance the membrane durability during PEMFC operation [10,11,12].

Among the methods designed to solve the drawbacks of aromatic hydrocarbon membranes, block copolymers having controlled morphology and a similar ion exchange capacity (IEC) value showed not only excellent mechanical behaviors but also high ionic conductivity when compared with the random copolymer.

Numerous studies related to the characterization of block copolymers have continuously improved the performance of electrolyte membranes applicable to PEM. Furthermore, the increase in repeat units of hydrophilic segments in the polymer affects the IEC value and effectively governs the aggregation of ion clusters. In another report, McGrath et al. [13] designed and synthesized multi-block copolymers containing sulfonated and fluorinated segments. They reported that the membranes have good thermomechanical stabilities and high ion conductivity in relation to commercial Nafion membrane under low humidity condition. Although the synthesis of block copolymer with controlled morphology has been found to be the most suitable strategy for producing a good performance of a fuel cell, still it has drawbacks such as difficult in controlling the molecular weight and limited application to commercialization.

Therefore, sulfonated bis[(4-chlorophenyl)sulfonyl]-biphenyl (BCPSBP) containing thiophene moiety as a hydrophilic oligomer can form steric hindrance, minimize interactions with radicals, and improve mechanical/thermal stabilities [14]. In addition, block copolymer containing hydrophobic oligomer with perfluorinated structure and hydrophilic oligomer is deduced as a strategy for the production of ideal PEM through lowering the volume change by water drift.

In line with these facts, the sulfonated poly(arylene biphenylether sulfone)-poly(arylene ether) (SPABES-PAE) block copolymers in di- or tri-block structure were designed in order to improve membrane properties, and these copolymers were investigated for their comparison. Initially, sulfonated SPABES-PAE block copolymers were synthesized with SPABES oligomer as the hydrophilic part and PAE oligomer as the hydrophobic part via direct polymerization reaction. The chemical structure of prepared block copolymers was analyzed using ^1^H nuclear magnetic resonance (^1^H NMR), Fourier transform infrared spectroscopy (FT-IR), and gel permeation chromatography (GPC). The properties of SPABES-PAE membranes were widely studied in terms of water uptake, ion exchange capacity (IEC), thermal stability, oxidative stability, and proton conductivity.

## 2. Experimental

### 2.1. Materials

4,4’-(hexafluoroisopropylidene)diphenol (6F-BPA) and decafluorobiphenyl (DFBP) were received from Alfa Aesar. 4,4’-bis[(4-chlorophenyl)sulfonyl]-biphenyl (BCPSBP), anhydrous N,N’-dimethylacetamide (DMAc), anhydrous toluene, and dimethyl sulfoxide-d_6_ (DMSO-d_6_) were purchased from Sigma–Aldrich (Saint Louis, MI, USA). Others solvents were obtained from commercial companies.

### 2.2. Synthesis of Polymers

#### 2.2.1. Synthesis of Sulfonated Poly(Arylene Biphenylehter Sulfone), Poly(Arylene Ether), and Poly(Arylene Biphenylether Sulfone)

We have prepared a di-sulfonated BCPSBP monomer (sBCPSBPms) in meta positions to the sulfone groups, and the synthetic steps of sBCPSBPms was followed from a previously reported paper [14,15]. The sulfonated poly(arylene ether biphenyl sulfone) (SPAEBS) was synthesized by direct nucleophilic substitution polymerization using sBCPSBPms and 6F-BPA (Scheme S1a). In a typical polymerization, sBCPSBPms (2.00 g, 2.60 mmol), 6F-BPA (1.31 g, 2.87 mmol), K_2_CO_3_ (1.19 g, 5.73 mmol), 30 mL of DMAc, and 15 mL of toluene were put together into 100 mL round-bottomed flask equipped with a Dean–Stark trap under nitrogen gas. The solution was refluxed at 100 °C for 2 h, and then the solution was heated at 160 °C for 4 h to remove toluene. The solution was increased to 170 °C and kept for 24 h to complete the polymerization. The viscous solution was slowly poured into the deionized (DI) water/methanol (*v*/*v* = 2/5) to precipitate solid powder. The precipitated powder was washed with methanol several times, filtered, and dried at 70 °C for 24 h. ^1^H NMR (600 MHz, DMSO-*d_6_*): 7.0–8.4 ppm; GPC (LiBr in dimethylformamide (DMF), refractive index (RI) detector) *M_n_* (number average molecular weight) = 20.9 kDa, *M_w_* (weight average molecular weight) = 41.3 kDa, polydispersity index (PDI) (*M_w_*/*M_n_*) = 2.0.

The fluorinated poly(arylene ether) (PAE) was prepared from 6F-BPA and DFBP (Scheme S1b) as described below. A 100 mL round-bottomed flask equipped with condenser was charged with 6F-BPA (2.00 g, 5.95 mmol), DFBP (2.21 g, 6.50 mmol), K_2_CO_3_ (1.81 g, 1.31 mmol), and DMAc (25 mL) under nitrogen gas. The mixture was stirred for 2 h at 110 °C and then the reaction temperature was raised 145 °C. The polymerization reaction was maintained for 24 h. After the polymerization reaction was maintained for 24 h, the mixture was cooled at room temperature (RT). The viscous solution was directly poured into DI water/methanol (*v*/*v* = 2/1) to produce the polymer powder. The polymer powder was collected by washing with methanol several times, filtered, and dried at 60 °C for 12 h. ^1^H NMR (600 MHz, DMSO-*d_6_*): 7.0–8.4 ppm; GPC (LiBr in DMF, RI detector) *M_n_* = 9.3 kDa, *M_w_* = 56.0 kDa, PDI (*M_w_*/*M_n_*) = 6.0.

The poly(arylene biphenyl ether sulfone) (PABES) oligomer was prepared using BCPSBP monomer following a similar method as described above. ^1^H NMR (600 MHz, DMSO-*d_6_*): 7.0–8.4 ppm; GPC (LiBr in DMF, RI detector) *M_n_* = 15.8 kDa, *M_w_* = 31.5 kDa, PDI (*M_w_*/*M_n_*) = 2.0.

#### 2.2.2. Synthesis of Block Copolymers (PABES-PAE and SPABES-PAE)

The PABES-PAE block copolymer was synthesized by nucleophilic substitution reaction using PABES and PAE to compare chemical properties with SPABES-PAE block copolymers, and the synthetic step is shown in Scheme S2. The polymerization procedure of PABES-PAE is as follows. A 100 mL round-bottomed flask equipped with Dean–Stark trap was charged with PABES oligomer (1.50 g, 3.00 mmol), PAE oligomer (1.10 g, 3.30 mmol), K_2_CO_3_ (1.10 g, 3.30 mmol), and DMAc/toluene (30 mL/20 mL). The mixture was heated to 130 °C for 4 h to remove toluene and moisture. After finishing the azeotrope, the mixture temperature was maintained at 170 °C for 48 h. After the reaction time, the viscous solution was poured in co-solvent (methanol:DI water = 1:1) to collect the white powder. The produced powder was obtained by filtration, washed with methanol several times, dried in oven at 80 °C for 24 h. ^1^H NMR (600 MHz, DMSO-*d_6_*): 7.0–8.4 ppm. ^1^H NMR (600 MHz, DMSO-*d_6_*): 7.0–8.4 ppm; GPC (LiBr in DMF, RI detector) *M_n_* = 14.8 kDa, *M_w_* = 122.8 kDa, PDI (*M_w_*/*M_n_*) = 8.3.

The SPABES-PAE block copolymers were prepared by controlling SPABES and PAE molar ratio (1:2, 1:1, and 2:1). The polymerization of SPABES-PAE block copolymer (1:1) is as follows. SPABES oligomer (1.00 g, 0.024 mmol), PAE oligomer (1.36 g, 0.024 mmol), K_2_CO_3_ (0.006 g, 0.048 mmol), and DMAc/toluene (25 mL/20 mL) were put into 100 mL round-bottomed flask with a Dean–Stark trap under an N_2_ atmosphere. Next, the reaction procedure and recrystallizations were carried out in a similar procedures to the preparation of PAE oligomer to collect SPABES-PAE block copolymer (1:1). ^1^H NMR (600 MHz, DMSO-*d_6_*): 7.0–8.4 ppm; GPC (LiBr in DMF, RI detector) *M_n_* = 13.9 kDa, *M_w_* = 96.5 kDa, PDI (*M_w_*/*M_n_*) = 6.9.

SPABES-PAE block copolymer (1:2). ^1^H NMR (600 MHz, DMSO-*d_6_*): 7.0–8.4 ppm; GPC (LiBr in DMF, RI detector) *M_n_* = 24.0 kDa, *M_w_* = 122.7 kDa, PDI (*M_w_*/*M_n_*) = 5.1.

SPABES-PAE block copolymer (2:1). ^1^H NMR (600 MHz, DMSO-*d_6_*): 7.0–8.4 ppm; GPC (LiBr in DMF, RI detector) *M_n_* = 16.7 kDa, *M_w_* = 112.3 kDa, PDI (*M_w_*/*M_n_*) = 6.7.

### 2.3. Membrane Preparation

The membrane of SPABES-PAE block copolymers was prepared by solution casting method. The copolymer solutions was casted on a glass dish and then dried in an oven at 90 °C for 24h. After, all membranes were acidified in 1 M H_2_SO_4_ solution at 95 °C for 3 h and washed several times to remove the excess H_2_SO_4_ over 10 h. Before using the membranes for measurement of chemical and electric properties, SPABES-PAE block copolymer membranes were obtained by drying in an oven at 90 °C for 12 h (membrane thickness was 70 ± 10 μm).

### 2.4. Chraterizations

The ^1^H NMR spectra were analyzed by JNM-ECA 600 instrument (JEOL, Akishima, Tokyo, Japan). The IR spectra were obtained by Frontier MIR/NIR spectrometer (PerkinElmer, Waltham, MA, USA). The molecular weights (*M_n_*, *M_w_*, and *M_z_*) and polydispersity indices (PDI, *M_w_*/*M_n_*) were measured by HLC-8320 (Tosoh Corporation, Minato, Tokyo, Japan). The thermogravimetric analysis (TGA) and differential scanning calorimetry (DSC) were conducted by Q50 and Q20 (TA Instruments, New Castle, DE, USA), respectively. The tapping mode-atomic force microscopy images were conducted by a Veeco multimode atomic force microscope (AFM, Veeco Corporation, Plainview, NY, USA). The proton conductivity was measured by conductivity test bench (Scitech Korea, Gangbuk, Seoul, Korea) [16,17,18]. The average ion domain size was recorded using EMPYREAN (Malvern Panalytical Ltd., Malvern, Grovewood, UK) [19,20]. The fuel cell performance was measured using a single cell test station (Scitech, Gangbuk, Seoul, Korea).

The activation energy (*E_a_*) of membranes was calculated using:

ln_σ_ = ln_σo_ − (*E_a_*/R × T), (1)

where R and T are the gas constant and Kelvin temperature, respectively [21].

The solubility of the copolymers was investigated by using concentration of 10 wt % at 60 °C in variety of solvents.

The oxidative stability of membranes was measured into 3% H_2_O_2_ containing 4 ppm FeSO_4_ solution (Fenton’s reagent) at 60 °C for 8 h [12,22].

The IEC of all membranes was measured by the titration method [15,22]. In brief, all acidified membranes were converted to salt form by immersing in 2 M NaCl solution for 36 h at room temperature. And then the solution was titrated with 0.1 N NaOH solution using phenolphthalein as an indicator.

The water uptake and swelling ratio was evaluated using dried and wet membrane [23,24]. The water uptake value of the membranes was reported using the following equation:

Water uptake (%) = (W_wet_ − W_dry_)/W_dry_ × 100%, (2)

where W_dry_ and W_wet_ are the membrane weights of the dried and wetted states.

The dimensional ratio was calculated using this equation:

Swelling ratio (%) = (S_wet_ − S_dry_)/S_dry_ × 100%, (3)

where S_wet_ and S_dry_ are the corresponding volumes of membrane length, width, and thickness, respectively [25,26].

### 2.5. Preparation of Membrane Electrode Assembly

The MEA was fabricated with membrane and commercial gas diffusion electrodes (GDE, loading of 0.3 mg·cm^–2^ Pt/C). The electrodes were sandwiched to both sides of the membrane (press condition: 110 °C, at 1400 psi for 1 min). The single cell active area was 5 cm^2^, and humidified H_2_ (anode flow rate: 0.1 L·min^–1^) and O_2_ (cathode flow rate: 0.4 L·min^–1^) were fed into the test station as fuel without any back pressure.

## 3. Results and Discussion

### 3.1. Synthesis and Structural Properties of SPABES-PAE Block Copolymers

**Figure 1:**
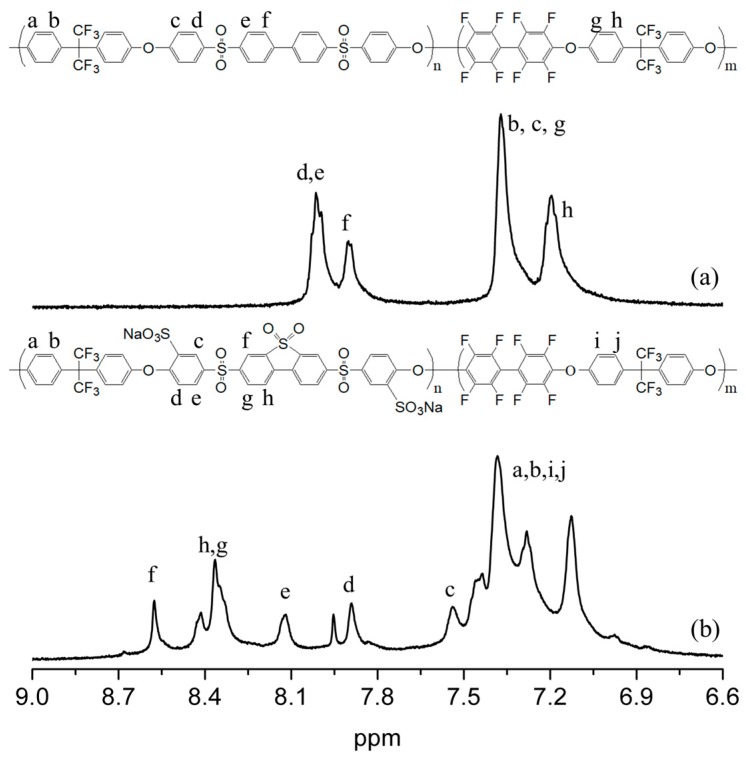
The proton signal of (**a**) poly(arylene biphenylether sulfone)-poly(arylene ether) (PABES-PAE) and (**b**) sulfonated poly(arylene biphenylether sulfone)-poly(arylene ether) (SPABES-PAE, 2:1) block copolymer.

The SPABES-PAE block copolymers were successfully synthesized with different molar ratios of SPABES and PAE oligomers through aromatic nucleophilic substitution reaction, and the synthetic step is shown in Scheme S1. The chemical structure of prepared copolymers were characterized by ^1^H NMR (Figure 1).

**Figure 2:**
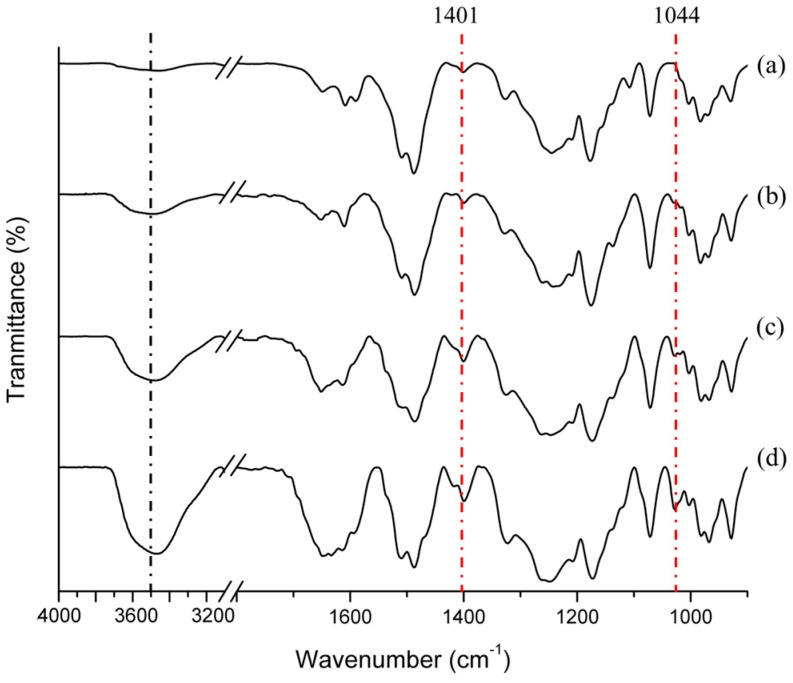
The IR spectra of block copolymers; (**a**) PABES-PAE, (**b**) SPABES-PAE (1:2), (**c**) SPABES-PAE (1:1), (**d**) SPABES-PAE (2:1).

The proton signal of the non-sulfonated PABES-PAE was exhibited at 8.0–7.0 ppm, which indicated the presence of phenylene groups. Whereas the proton signal of SPABES-PAE block copolymer was shown at 8.7–7.0 ppm, and this observation coincided with the results from a previously reported paper [12,15,25]. The proton signal of ortho to sulfone was shifted to the downfield owing to the strong electron-withdrawing sulfonic acid groups. This tendency was also observed in the IR absorbance (Figure 2). The IR absorbance of the diphenyl sulfone unit (split symmetric vibration) was observed at 1013 and 1074 cm^–1^. The absorption bands of C=C stretching vibration was assigned at 1508 and 1595 cm^–1^, and the absorption band of the ether (C–O–C) was assigned at 1072 cm^–1^. The intensity of the characteristic stretching vibrations of the sulfonic acid group also increased at 1044 cm^–1^ and 1401 cm^–1^, as the molar ratio of SPABES oligomer increased [15,27]. The molecular weights (*M_w_* and *M_n_*) and PDI of copolymers are shown in Table 1 (molecular weights of the non-sulfonated PABES-PAE and oligomers is shown in Table S1). The *M_w_* of SPABES-PAE block copolymers (1:2, 1:1, and 2:1) was in the range of 96 kDa to 122 kDa, specifying well formation of di- and tri- block copolymers. To investigate the solubility of polymers, the solubility was evaluated using a variety of solvents with a concentration of 10 wt % polymer, and is presented in Table S2. As a result, SPABES copolymers were easily dissolved in several polar aprotic solvents, and were not dissolved in polar protic solvents.

### 3.2. Thermal Stabilities

**Figure 3:**
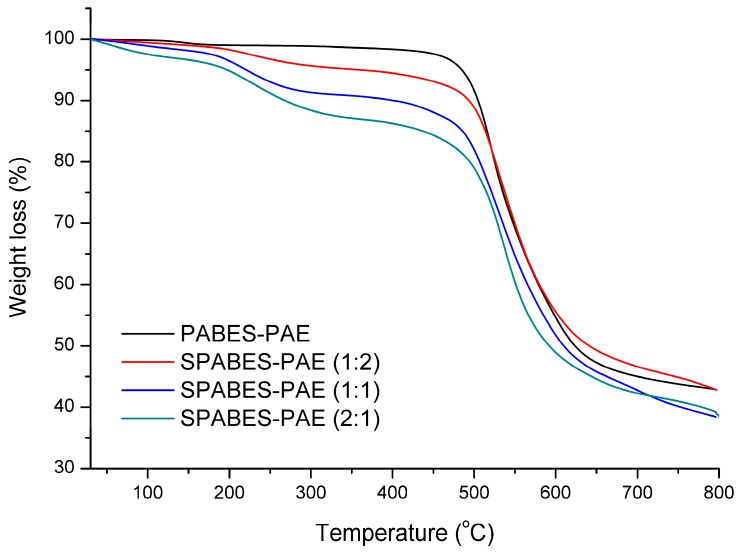
Thermogravimetric curves of the block copolymers measured at 10 °C·min^−1^ under nitrogen flow.

Thermogravimetric analysis results of SPABES copolymers are given in Figure 3. The TGA patterns of all copolymers exhibited three-step weight losses, except the PABES copolymer, as shown Figure 3. The initial decomposition of SPABES-PAE block copolymers between 50 and 150 °C is associated with the removal of non-bound and bound water molecules in the membranes. The second decomposition of SPABES copolymers between 250 and 350 °C is related with the elimination of sulfonic acid groups [28,29,30]. The third decomposition approximately over 420 °C is ascribed to the ruining of aromatic polymer backbones. Differential scanning calorimetry results of the SPABES copolymers are presented in Table 2 and Figure S1.

From these results, it can be seen that the glass transition (T_g_) value increases as the hydrophilic molar ratio increases. This may be due to the high glass transition temperature of sulfone bridges in the hydrophilic oligomer [16]. Altogether, the thermal stabilities of SPABES-PAE are sufficient to sustain the operation of fuel cells below 90 °C.

### 3.3. Water Uptake, IEC, and Hydration Number Subsection

Both the hydrophilic and hydrophobic domains have effects on the properties of membrane such as water absorption, dimensional stability, proton conductivity, etc. Generally, the sulfonated aromatic membranes possessing IEC values of over 2.0 mequiv.·g^−1^ show an excessive water uptake and swelling ratio. On the other hand, the aromatic polymer membrane possessing aggregation of the hydrophobic domain reduce the swelling ratio and water uptake, and the membranes containing fluorine functional groups in the backbone were reported to have well phase separation and high dimensional stability in the hydrated state [31]. The results of water uptake are presented in Table 3 and Figure S1a. As expected, the water uptake of SPABES-PAE membranes increased with the increasing molar ratio of the hydrophilic block. The water absorption of the SPABES-PAE membranes was 21%, 36%, and 52%, respectively. These results indicate that the SPABES-PAE membranes have low water uptake compared with the most recently reported PEMs [32,33,34,35] (Table 4). The IEC of Nafion 212 and SPABES-PAE membranes was in the range of 0.55–1.23 mequiv.·g^−1^. The IEC of SPABES-PAE membranes increased as the molar ratio of hydrophilic oligomer increased (Table 3). The hydration number of membranes was calculated from the results of IEC and water uptake. Generally, the hydration number indicates the number of water molecules absorbed per unit volume of the –SO_3_H group in the membranes. According to the evaluation result, Nafion 212 showed λ value of 25.6, while SPABES-PAE membranes (1:2, 1:1, and 2:1) showed λ values of 21.2, 22.7, and 23.6.

### 3.4. Dimensional Change and Mechanical Strength

Generally, the dimensional change of the membrane is related to the IEC value. The PEMs with high IEC lead to high water absorption, resulting in excessive dimensional changes [36,37]. Therefore, the dimensional stability of the membrane is one of the important key factors to be evaluated before fabrication of MEA. As shown in Table 3 and Figure S1b–d, the dimensional stability of SPABES-PAE and Nafion 212 membranes were in the range of 26.9–52.1% at 90 °C. Among the SPABES-PAE membranes, dimensional stability of SPABES-PAE (2:1) was 1.26 times lower than Nafion 212 membranes, which indicate that the PEMs containing strong hydrophobic parts improve the repulsive power against to water.

The PEMs have sufficient physical strength to be used in the fabrication of MEAs. The mechanical behavior (i.e., tensile strength (TS), Young’s modulus (YM), and elongation at break (EB)) of SPAE-PAE membranes was investigated at 25 °C and fully hydrated and are shown in Table 2. According to the results, TS and YB decreased while EB increased with increasing hydrophilic molar ratio. This means that as the hydrophilic molar ratio in the polymer increases, the flexibility and elasticity of the membrane increases.

### 3.5. Proton Conductivity and Activation Energy

**Figure 4:**
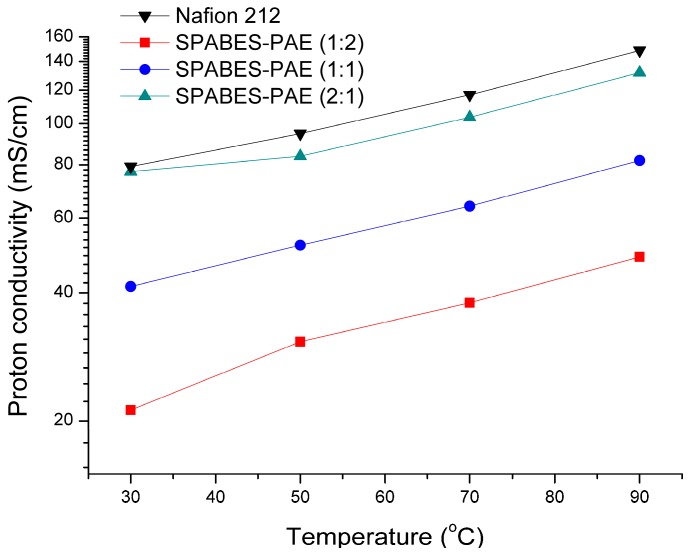
Proton conductivity of membranes at various temperature and 100% relative humidity (RH).

The proton conductivity values of the SPABES-PAE and Nafion 212 membranes are presented in Figure 4 and Table 5. The proton conductivity values of Nafion 212 and SPABES-PAE membranes increased with increasing temperature due to rapid proton movement. It can be seen that all the membranes showed proton conductivity of 48–132 mS·cm^−1^. The proton conductivity of SPABES-PAE (2:1) membrane was up to 132 mS·cm^−1^ at 90 °C, which is closer to the proton conductivity of Nafion 212 (148 mS·cm^−1^). This may be due to a higher number of proton conducting highways in SPABES-PAE membranes attained by a high ratio of hydrophilic oligomer [38,39,40]. As the hydrophilic oligomer content increased, the prepared SPABES-PAE membranes provided a broad pathway for proton conduction. Then, broad proton conduction pathways increase the movement of protons through the membranes.

**Figure 5:**
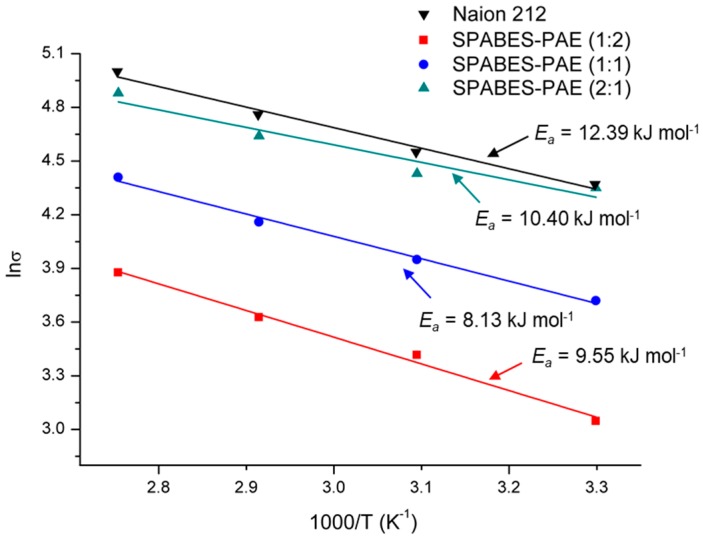
Arrhenius plot of membranes at various temperature and 100% RH.

The calculated activation energies of SPABES-PAE membranes (1:2, 1:1 and 2:1) were 10.89, 10.39, and 9.22 kJ·mol^−1^ (Figure 5 and Table 4) [41], respectively, while that of Nafion 212 was 9.56 kJ·mol^−1^. According to the results, the activation energies of SPABES-PAE and Nafion 212 membranes were low, indicating that proton transport is easy in the prepared membranes and Nafion 212 because low activation energies mean low membrane resistance.

### 3.6. Oxidative Stability and Single Cell Performance

**Figure 6:**
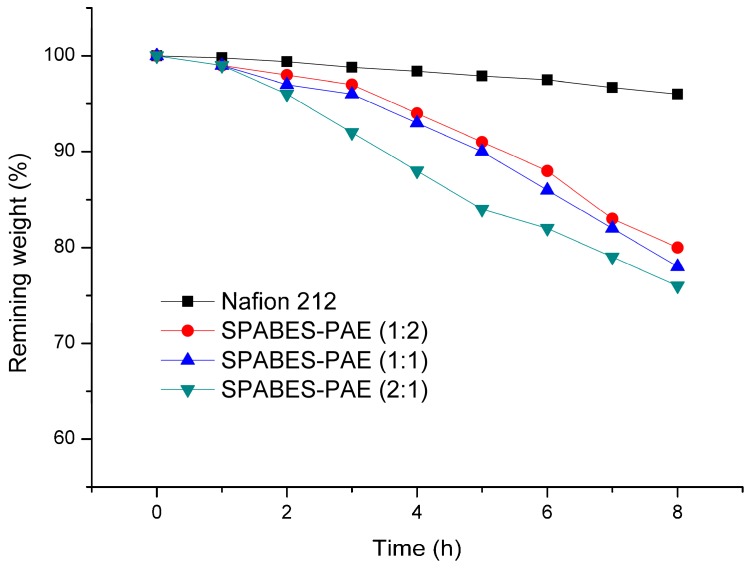
Accelerated oxidative stability test in 4 ppm Fe^2+^ Fenton’s reagent at 60 °C.

The oxidative stability of fabricated membranes was investigated at 60 °C in Fenton’s reagent (4 ppm FeSO_4_ in 3% H_2_O_2_) and the results are summarized in Figure 6. The oxidative stability of membranes was measured for 8 h in Fenton’s reagent. The weight loss (%) of SPABES-PAE membranes was slightly changed over 1 h. However the decomposition of prepared membranes exhibited sharp increases after 1 h, and the residual weight (%) of SPABES-PAE membranes (1:2, 1:1, and 2:1) were 80%, 78%, and 76% after 8 h, respectively. The better stability of SPABES-PAE membrane compared to other PEMs can be ascribed to the block copolymer structure containing fluorine and thiophene units, which indicated that the fluorine and thiophene units enhanced the resistance of radical attack, and these results well matched with previous reported papers [15,42,43].

**Figure 7:**
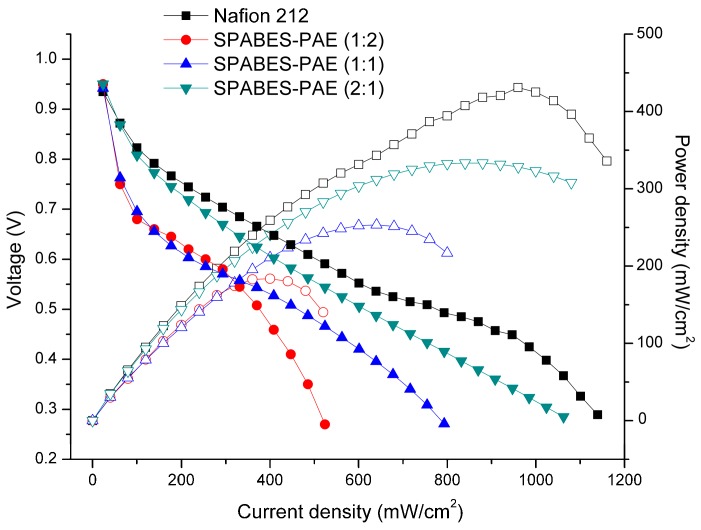
Fuel cell performance of membranes at 60 °C under 100% RH.

All MEAs were fabricated with SPABES-PAE and Nafion 212 membranes to obtained polarization and open circuit voltage (OCV) curves. The polarization and power density curves of SPABES-PAE and Nafion 212 membranes were investigated under 100% RH at 60 °C and the results are shown in Figure 7 and Table 5. All prepared membranes exhibited an OCV around 0.92 V, indicating that the membranes had good gas barrier properties in environments of H_2_ and O_2_ gas systems [44,45,46]. The maximum power density of fabricated MEA of SPABES-PAE showed a difference depending on hydrophilic oligomer ratio in polymer. The maximum power density of SPABES-PAE membranes (1:2, 1:1, and 2:1) were 183.6, 253.3, and 333.29 mW·cm^−2^, respectively. Although Nafion 212 when compared to prepared SPABES membranes showed the best single cell performance, the power density of the SPABES X2Y1 membrane confirmed that the results were very close to Nafion 212 (430.9 mW·cm^−2^). The SPABES-PAE membrane is expected to be a very promising membrane to replace Nafion 212. Therefore, these results clearly indicate that the hydrophilic oligomer ratio in the polymer membrane increased the electrochemical properties, due to the retention of large amounts of water molecules and increase of ion channels.

### 3.7. Morphology

**Figure 8:**
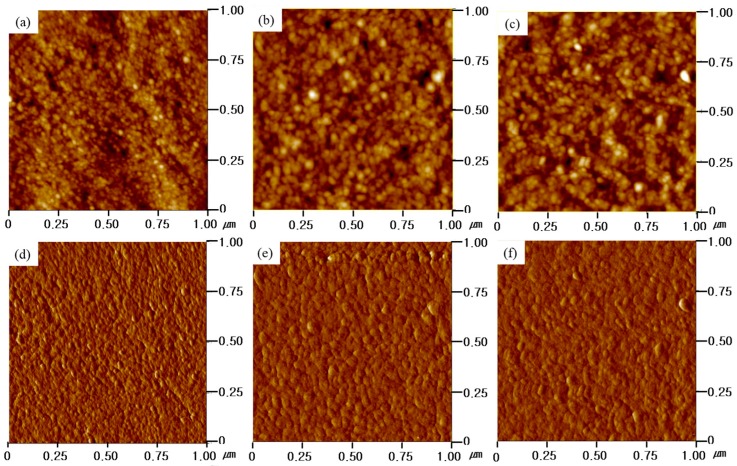
Atomic force microscopy (AFM) height images of membranes of (**a**) SPABES-PAE (1:2), (**b**) SPABES-PAE (1:1), and (**c**) SPABES-PAE (2:1); AFM phase images of membranes of (**d**) SPABES-PAE (1:2), (**e**) SPABES-PAE (1:1), and (**f**) SPABES-PAE (2:1).

**Figure 9:**
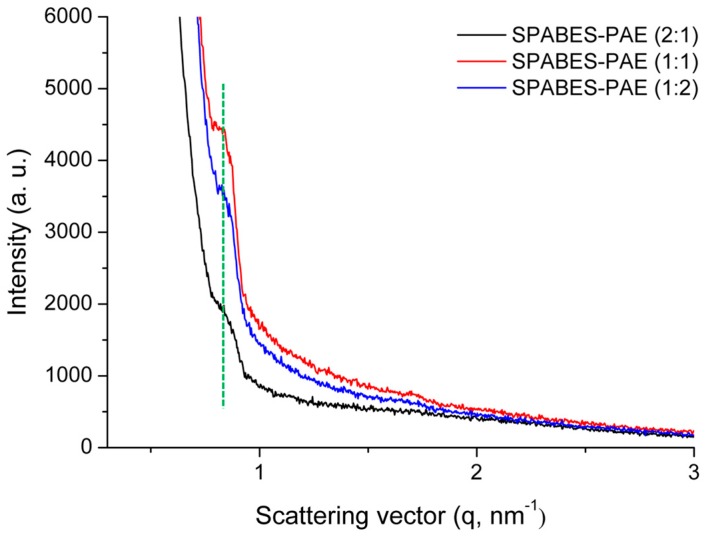
The SAXS patterns of SPABES-PAE membranes under a fully hydrated state.

The microstructure of the SPABES-PAE membranes was investigated by atomic force microscopy (AFM) (Figure 8). In this AFM phase image, the bright regions represent the hydrophobic domains, whereas dark regions represent the hydrophilic domains [47,48,49]. The AFM image suggests that the sulfonic acid groups were aggregated, which provide a proton transfer pathway or an ion transfer pathway. It was confirmed that as the hydrophilic oligomer content increased, the dark domain areas increased, and this suggests that the ionic conductivity increased. Furthermore, the ion cluster size of SPABES-PAE membranes was analyzed by small angle X-ray scattering (SAXS) measurement, and the results are shown in Figure 9 and Table 6. The scattering vector (q) of SPABES-PAE membranes is shown in the range of 0.810–0.817 nm^−1^, which indicates the ionic cluster dimension of approximately 7.71 nm. Even if the SPABES-PAE membranes have different molar ratio of hydrophilic and hydrophobic oligomers, the SAXS pattern showed similar ion cluster size.

## 4. Conclusions

The SPABES-PAE (1:2, 1:1, and 2:1) block copolymers were synthesized by controlling the molar ratio of SPABES and PAE oligomers via a nucleophilic aromatic substitution reaction. The chemical structure of synthesized SPABES-PAE was confirmed through ^1^H NMR, IR, and GPC. The fabricated SPABES-PAE membranes have reasonable dimensional stabilities (water uptake and swelling ratio), and the AFM and SAXS results show well-defined ion transport channels. The proton conductivity of SPABES-PAE (2:1) membrane (IEC = 1.23 mequiv.·g^−1^) showed 129 mS·cm^−1^ (at 90 °C under 100% RH), which is similar to Nafion 212 (148.6 mS·cm^−1^, under the same condition). Moreover, the SPABES-PAE (2:1) membrane (333.2 mW·cm^−2^) showed remarkable sing cell performance at 100% RH and 60 °C, when compared with Nafion 212 (430.9 mW·cm^−2^). Overall, the SPABES-PAE membranes showed high oxidative/thermal stability compared with other aromatic hydrocarbon membranes, and balanced properties of PEM between water resistances and proton conductivities, which indicate that it is a promising candidate as PEM material for PEMFCs.

## Figures and Tables

**Table 1:** The molecular weights (*M_n_*, *M_w_*, and *M_z_*), and polydispersity index (PDI) of block copolymers.

Copolymer	*M*_n_ (kDa)	*M*_w_ (kDa)	*M*_z_ (kDa)	*M*_w_/*M*_n_ (PDI)
SPABES-PAE (1:2)	24.0	122.7	418.0	6.9
SPABES-PAE (1:1)	13.9	96.5	411.1	6.7
SPABES-PAE (2:1)	16.7	112.3	453.1	5.1

**Table 2:** The mechanical behaviors and glass transition temperature of block copolymers.

Copolymer	TS (MPa)	YM (GPa)	EB (%)	T_g_ (℃)
SPABES-PAE (1:2)	40.3	0.48	17.9	177
SPABES-PAE (1:1)	36.8	0.36	18.1	184
SPABES-PAE (2:1)	28.9	0.33	19.1	188
PABES-PAE (1:1)	48.4	0.48	4.8	194

Tensile strength (TS), Toung’s modulus (YM), elongation at break (EB), T_g_: glass transition temperature.

**Table 3:** Ion exchange capacity (IEC), water uptake, and dimensional stability of SPABES-PAE and Nafion 212 membranes.

Membrane	IEC (mequiv.·g^−1^)	Water Uptake (%)		Dimensional Stability (%)	
		30 °C	90 °C	30 °C	90 °C
SPABES-PAE (1:2)	0.55	14	21	3.0	26.9
SPABES-PAE (1:1)	0.87	24	36	4.5	39.3
SPABES-PAE (2:1)	1.23	34	52	10.1	42.4
Nafion 212	0.91	18	42	3.0	52.1

**Table 4:** IEC, water uptake, and proton conductivity comparison between recently reported proton exchange membranes (PEMs).

Membrane	IEC (mequiv.·g^−1^)	Water uptake (%) at 90 °C	Proton conductivity (mS·cm^−1^)	Refs
SPABES-PAE (1:2)	0.55	21.1	48.6 (90 °C)	this work
SPABES-PAE (1:1)	0.87	36	81.9 (90 °C)	this work
SPABES-PAE (2:1)	1.23	52.2	131.9 (90 °C)	this work
tsPTPO-100	1.65	72.1	96.4 (90 °C)	[32]
IBQSH-60	1.39	30.0	49.0 (80 °C)	[33]
SPAES-6FBPA-40	1.25	36.5	101.1 (90 °C)	[34]
B20V80-SDPA	1.89	65.1	114.4 (90 °C)	[35]

**Table 5:** Hydration number, proton conductivity, activation energy (*E_a_*), and power density of SPABES-PAE and Nafion 212 membranes.

Membrane	Hydration number ^a^ (H_2_O/SO_3_H)	Proton conductivity (mS·cm^−1^) ^b^		*E*a (kJ·mol^−1^)	Peak power density (mW·cm^−2^) ^c^
		30 °C	90 °C		
SPABES-PAE (1:2)	21.2	21.2	48.6	10.89	183.6
SPABES-PAE (1:1)	22.7	41.4	81.9	10.39	253.3
SPABES-PAE (2:1)	23.6	77.1	131.9	9.22	333.2
Nafion 212	25.6	79.3	148.6	9.56	430.9

^a^ Reference [13]. ^b^ Measured under 100% RH. ^c^ Measured at 60 °C under 100% RH.

**Table 6:** The scattering vector (q_max_) and ion cluster size of SPABES-PAE membranes.

Membrane	Scattering vector (q_max_, nm^−1^)	Ion cluster size (nm)
SPABES-PAE (1:2)	0.810	7.75
SPABES-PAE (1:1)	0.815	7.71
SPABES-PAE (2:1)	0.817	7.69

## Supplementary Materials

The following are available online at http://www.mdpi.com/2073-4360/10/12/1367/s1. Scheme S1: The synthetic step of SPABES-PAE block copolymer; Scheme S2: The synthetic step of PABES-PAE block copolymer; Figure S1: The DSC analysis of the membranes measured at 10 °C·min^−1^ under N_2_ flow; (**a**) SPABES-PAE (1:2), (**b**) SPABES-PAE (1:1), (**c**) SPABES-PAE (2:1), and (**d**) PABES-PAE (1:1); Figure S2: The (**a**) water uptake and (**b**–**d**) swelling ratio (length, width, and thickness) of SPABES-PAE and Nafion 212 membranes at various temperature; Table S1: The molecular weights (*M_n_*, *M_w_*, and *M_z_*), and PDI of copolymers; Table S2: Solubility behavior of the SPABES copolymers in various solvents.
