# Supplementary material for: Facile Fabrication and Characterization of Improved Proton Conducting Sulfonated Poly(Arylene Biphenylether Sulfone) Blocks Containing Fluorinated Hydrophobic Units for Proton Exchange Membrane Fuel Cell Applications
Source: Polymers (Basel). 2018 Dec 10;10(12):1367. doi: 10.3390/polym10121367 (PMC6401750; doi:10.3390/polym10121367)
Supplement: Supplementary file 1 [file polymers-10-01367-s001.pdf]

polymers

MDPI

Article

Facile Fabrication and Characterization of Improved Proton Conducting Sulfonated Poly(Arylene Biphenylether Sulfone) Blocks Containing Fluorinated Hydrophobic Units for Proton Exchange Membrane Fuel Cell Applications

Kyu Ha Lee [1], Ji Young Chu [1], Ae Rhan Kim [2,*], Dong Jin Yoo [1,3,*]

1 Department of Energy Storage/Conversion Engineering of Graduate School, Hydrogen and Fuel Cell Research Center, Chonbuk National University, Jeonju 54896, Republic of Korea; carumiss@naver.com (K.H.L.), ebbuneg@hanmail.net (J.Y.C)
2 R&D Center for CANUTECH, Business Incubation Center and Department of Bioenvironmental Chemistry, Chonbuk National University, Jeonju 54896, Republic of Korea;
3 Department of Life Science, Chonbuk National University, Jeonju 54896, Republic Korea;
* Correspondence: canutech@hanmail.net (A.R.K), djyoo@jbnu.ac.kr (D.J.Y)

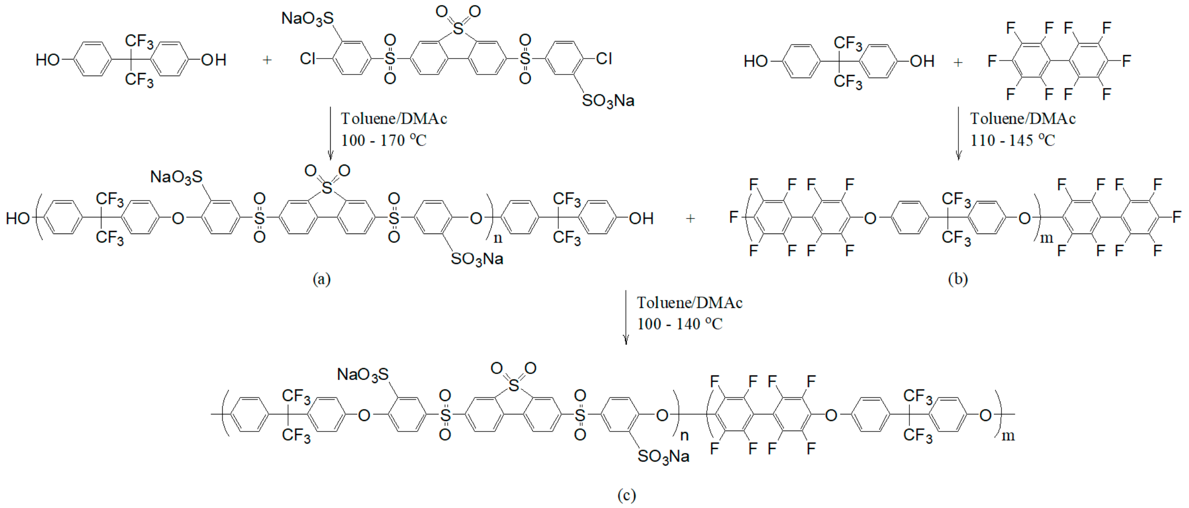

Scheme S1. The synthetic step of SPABES-PAE block copolymer.

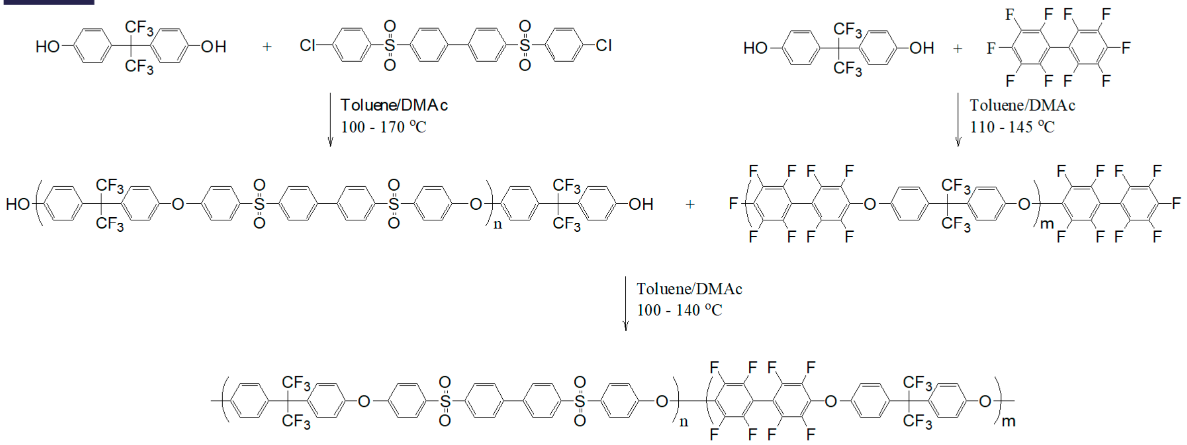

Scheme S2. The synthetic step of PABES-PAE block copolymer.

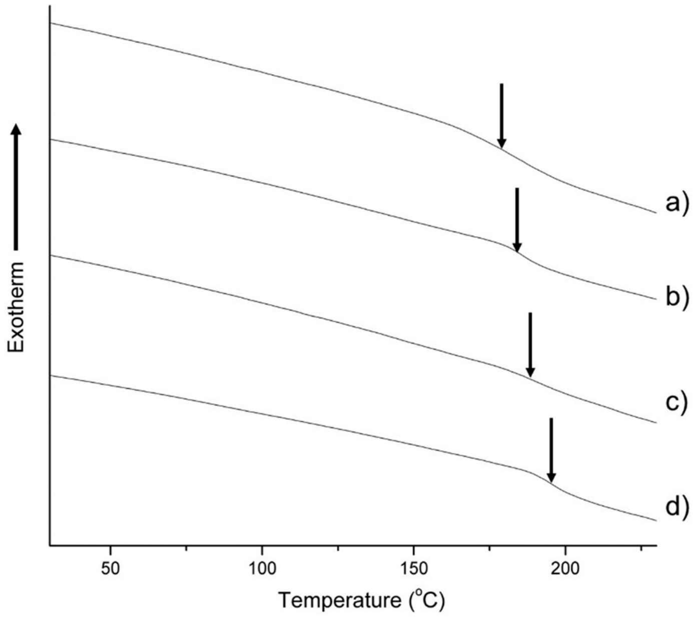

Figure S1. The DSC analysis of the membranes measured at 10 °C min^{-1} under N_2 flow; a) SPABES-PAE (1:2), b) SPABES-PAE (1:1), c) SPABES-PAE (2:1), and d) PABES-PAE (1:1).

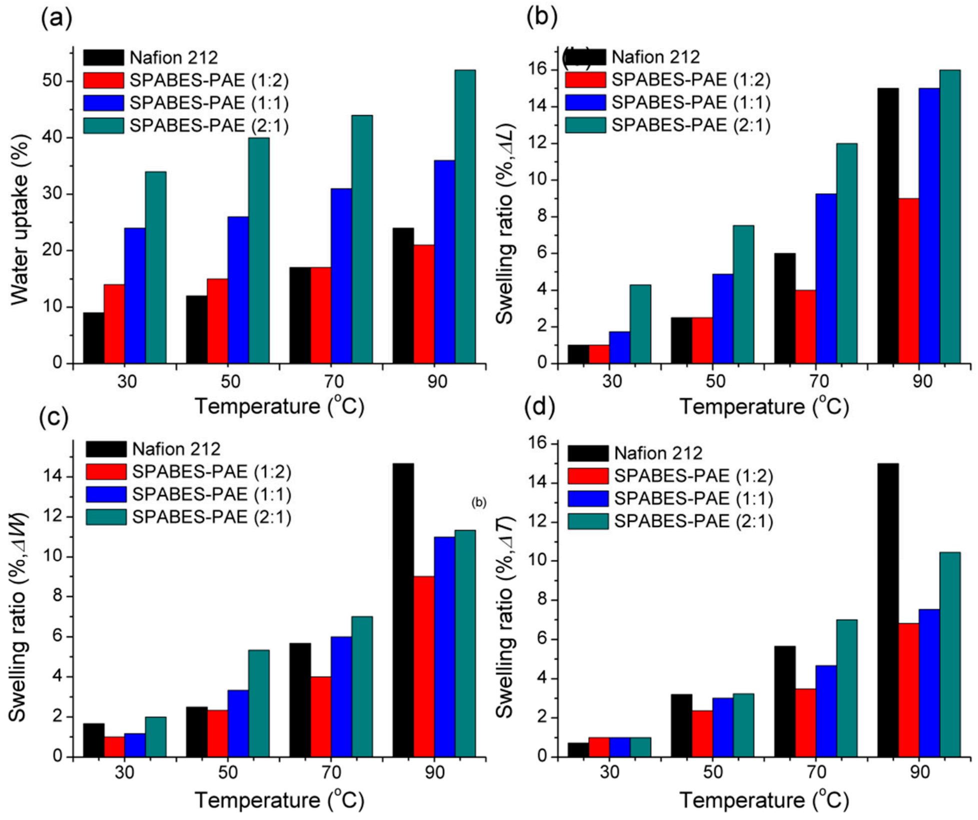

Figure S2. The (a) water uptake and (b-d) swelling ratio (length, width, and thickness) of SPABES-PAE and Nafion 212 membranes at various temperature.

polymers
MDPI

Table S1. The molecular weights (M_n, M_w, and M_z), and PDI of copolymers.

Copolymer	M_n (kDa)	M_w (kDa)	M_z (kDa)	M_w/M_n (PDI)
SPABES oligomer	20.9	41.3	76.7	2.0
PAE oligomer	9.3	56	109.1	6.0
PABES oligomer	15.8	31.5	68.4	2.0
PABES-PAE (1:1)	14.8	122.8	574.3	8.3

Table S2. Solubility behavior of the SPABES copolymers in various solvents.[a]

Copolymer	SPABES-PAE (1:2)	SPABES-PAE (1:1)	SPABES-PAE (2:1)
DMSO	++	++	++
NMP	++	++	++
DMAc	+	+	+
DMF	+	+	+
THF	–	–	–
Chloroform	–	–	–
Acetone	–	–	–
Methanol	–	–	–
Water	–	–	–

[a] **(++) highly soluble, (+) soluble, (–) insoluble at 60** °C
